# Achieving health-promotion practice in primary care using a multifaceted implementation strategy: a non-randomized parallel group study

**DOI:** 10.1186/s43058-025-00723-y

**Published:** 2025-04-07

**Authors:** Ylva Elisabet Nilsagård, Daniel Robert Smith, Fredrik Söderqvist, Emma Nilsing Strid, Lars Wallin

**Affiliations:** 1 University Health Care Research Centre, Faculty of Medicine and Health, Örebro University, 701 82 Örebro, Sweden; 2 Department of Epidemiology and Biostatistics, Faculty of Medicine and Health, Örebro University, 701 82 Örebro, Sweden; 3 Department of Health and Welfare, Dalarna University, 791 88 Falun, Sweden

**Keywords:** Primary Health Care, Health Promotion, Healthy Lifestyle, Implementation Science, Change Management, Clinical Practice Guidelines

## Abstract

**Background:**

Evidence-based healthcare recommendations exist for tobacco use, harmful alcohol consumption, low physical activity, and poor diet. However, the uptake of these recommendations in Swedish primary healthcare is poor, and the potential benefits for patients are not fully realized. Our aim was to evaluate the effect (i.e. the uptake) of a 12-month multifaceted implementation strategy to achieve a more health-promoting practice. We hypothesized that primary healthcare centers receiving this strategy would increase and sustain their health-promotion practices to a significantly greater extent than control centers, from baseline to the 6-month follow-up.

**Methods:**

In a non-randomized parallel group study, 5 intervention centers and 5 matched control centers were compared regarding health-promotion activities delivered in relation to visits to each center. The intervention centers received a multifaceted implementation strategy over at least 12 months based on established strategies, the Astrakan model of leading change, and findings from pre-implementation studies. The main strategies were: using external and internal facilitators to combine bottom-up and top-down perspectives, and emphasizing leadership responsibility for change. Medical record data on health-promotion activities, including prescribed physical activity and use of lifestyle screening forms, were collected monthly for 2 years: 6 months before and after implementation, and during the implementation phase. The implementation strategy effect was estimated using generalized linear mixed models.

**Results:**

During the 12-month implementation phase, the intervention and control sites had 135 002 and 160 987 healthcare visits, respectively; conducted 8839 and 6171 health-promotion activities, respectively; and administered 2423 and 282 lifestyle screening forms, respectively. A statistically significant higher relative uptake rate of health-promotion activities was found in intervention sites compared to control sites after the implementation period compared to before. The effect increased during the active phase, with the intervention sites having on average 1.07 and 2.0 times the uptake rate of the control sites at 1 and 12 months, respectively; this effect was largely maintained during the 6-month post-intervention phase. A significant absolute effect, in terms of difference in predicted uptake per 1000 visits, was evident 7 months into the implementation phase.

**Conclusion:**

This multi-faceted implementation strategy was successful in achieving a more health-promoting practice.

(ClinicalTrials.gov ref: NCT04 799,860, 03/04/2021, https://clinicaltrials.gov/study/NCT04799860).

**Trial registration:**

This study is part of the Act in Time project, registered at ClinicalTrials.gov on 4 March 2021 (ref: NCT04 799,860).

**Supplementary Information:**

The online version contains supplementary material available at 10.1186/s43058-025-00723-y.

Contributions to the literature

- This study presents an implementation strategy targeting all professionals who encounter patients in primary healthcare centers.
- The implementation strategy integrated a model for leading change to provide structure for working inclusively, promoting autonomy, and enabling competence building
- The study used a combined bottom-up and top-down approach that seems promising for scaling up health-promotion practice.

## Background

There is an ongoing paradigm shift in Swedish healthcare, from a healthcare system built up around diseases and institutions towards a more people-oriented one. The demographic challenge is that there will be fewer people available to provide care for people who are living longer with chronic and often preventable diseases. Healthy lifestyle habits can prevent occurrences of cardiovascular disease and stroke as well as 30% of all cancer [1, 2], and can prevent or delay the development of type 2 diabetes [3]. Individuals who have already developed these diseases can also gain large health benefits by changing to more healthy habits [4]. A person who stops smoking, starts to eat more healthily, and takes up regular exercise after an acute coronary artery event may after only 6 months reduce their risk of new cardiac events by 74% compared to a person who continues to smoke, remains physically inactive, and does not improve their dietary habits. A shift from reactive to proactive practice is needed to meet future demands. In line with this reform, primary healthcare centers (PHCCs) need to provide a more health-promoting practice, using individualized lifestyle interventions to address the increase in these noncommunicable diseases that remain the leading cause of disability and premature death [5, 6]. Health promotion is the process of empowering people to control and improve their health [7]. This implies an approach that strengthens and improves health knowledge, attitudes, skills, and behaviors from both population and individual perspectives.

Healthcare providers are obliged to inform patients about methods that prevent disease, according to the Patient Act [8], and the National Guideline for the Prevention and Treatment of Unhealthy Lifestyles provides low-risk, evidence-based recommendations of interventions for tobacco use, harmful use of alcohol, low physical activity, and poor diet [9]. These recommendations include information on how and when to provide advice or refer the patient for more in-depth counseling, and the use of specific codes for medical record entries for each activity.

It is widely acknowledged that the uptake of evidence-based recommendations is slow and incomplete [10, 11], and the World Health Organization has stated that one of this century’s most important public health challenges is to bridge the gap between knowing and doing [12]. In Sweden, health-promotion work in primary healthcare (PHC) increased between 2013 and 2019, although there was a break in this trend when the COVID-19 pandemic began [13]. Although PHCCs are required to provide health-promotion activities, the uptake in clinical practice is poor and the potential benefits are not fully realized. Guidelines alone rarely drive change, but adoption requires varying degrees of change in practice behavior.

There is a knowledge gap about how to support staff, regardless of profession, to achieve a more health-promoting practice in PHCCs. We have previously reported perceived barriers and facilitating factors from the perspective of managers, staff, and appointed internal facilitators (IFs) at a pre-implementation stage in designing the implementation strategy used in the present study [14]. This previous work highlighted the necessity of a collective understanding of the purpose of changing to more health-promoting practices. PHC staff shared beliefs and knowledge about the impact of health-promotion work in facilitating change, while lack of competence, structure, and time, as well as low cooperation between professionals, were described as barriers. Staff also expressed the need for clear direction at each management level to increase health promotion. Managers at all levels expressed that they needed practical support from higher levels of management to lead and enable the change, as well as help to prioritize health promotion [15].

It is of utmost importance to find effective ways to support proactive healthcare and thereby slow down or reverse the increase in illness due to unhealthy lifestyles. The present study will provide further knowledge on the uptake of health-promotion practice using a multifaceted implementation strategy in PHC, and hence guide future implementation efforts.

## Methods

### Aim

The aim was to evaluate the effect (i.e. the uptake) of a 12-month multifaceted implementation strategy to achieve more health-promoting practice in a PHC setting. We hypothesized that PHCCs that received the implementation strategy would increase and sustain their health-promotion practices to a significantly greater extent than the control centers, from baseline to 6-month follow-up.

The specific research questions were:

Will the rate of individualized health-promotion activities increase more in the intervention sites than in the control sites during the implementation phase and at the 6-month follow-up, as shown by administrative registration of simple advice, consultative conversation or qualified consultative conversation and physical activity prescriptions?

Will staff at the intervention PHCCs send proportionally more lifestyle screening forms to patients with questions targeting tobacco use, harmful use of alcohol, low physical activity, and poor diet compared to staff at the control sites during the implementation phase and the 6-month follow-up phase?

### Design

A non-randomized parallel group study design was used to compare intervention and matched control centers with respect to health-promotion interventions registered in medical records [16]. A pre-post design is useful in examining the impact of a complex implementation strategy in a real world setting when a randomized controlled trial is not feasible, and when assessing the adoption and adherence to guideline recommendations by the healthcare systems [17, 18]. A prerequisite for the project was the willingness and readiness of the centers to change the way they worked [19]. Intervention and control centers were enrolled between May and December 2021. Data were collected over at least 24 months for each center, excluding holiday months with reduced staffing.

### Setting and context

The study included one third of the 28 PHCCs located in Region Örebro, which has approximately 307,000 inhabitants. The PHC constitute the base of Swedish healthcare and is where the citizens primarily should seek care. The requirements on content and quality are the same for all PHCCs within the region. Taxes and governmental contributions are the main funding source for the Swedish healthcare system. The centers are financed according to a per-capita reimbursement model, with some adjustments for the population profile in each service area. Of interest for the present study is the reimbursement for providing qualified support to quit smoking (goal: ≥ 3/1000 listed) and prescribed physical activity (goal: 8/1000 listed).The overarching operational plans for the Healthcare Administration (HCA) in Region Örebro (2022–2024) state that good health is important for the population, but give no directions on how to achieve this. The HCA, which is responsible for reimbursement and quality in PHC, declares that all PHCCs should work with systematic health promotion and disease prevention, and that the work emanating from the national guideline on tobacco use, alcohol habits, physical activity, and food habits should be strengthened. At study start, the prerequisites for all PHCCs were equal. During the last twenty years, almost all PHCCs have had appointed professionals with the responsibility to be updated regarding tobacco and physical activity as part of their work. Since 2022, the PHCCs have also appointed professionals to be updated regarding alcohol and diet recommendations. Usual care was provided at the matched controls. The Unit of Development started a network to gather all appointed professionals in 2022. Information sheets with patient information focusing on the respective lifestyle habit and screening forms were available digitally for all PHCCs.

### Targeted PHC centers

After informing all PHCC managers in the region, we consecutively recruited centers as intervention centers. The inclusion criterion was that the manager was interested in trying to achieve a change and was willing to identify 2–3 staff members who could set aside 4 h/week to act as IFs. Five centers fulfilled these criteria. In dialogue with the Advisory Board, which consisted of the Chief Executive Officer (CEO) and the vice CEO of the Integrated Healthcare Administration (IHA) and the CEO of the HCA, we identified five centers matched on socioeconomic status and location (rural vs. urban) to serve as controls. The project leader (Y.N.) met with the Advisory Board about three times a year during the study period.

The clinical intervention included the following steps:

1. Encourage patients to complete a standardized lifestyle screening form (digitally or at the PHCC) prior to their scheduled visit.
2. Invite those with identified unhealthy lifestyle habits to individualized simple or qualified lifestyle advice or counseling according to the national guidelines and provide follow-up when called for.
3. Document health-promotion activities in the medical record using specific codes.

The 12-month multifaceted implementation intervention was aimed at changing behavior in healthcare workers meeting patients. The intervention was based on the Astrakan leadership change model [20], which includes four phases. In the first phase, the aim is to gain an understanding of the current state: to define what the problem or opportunity is, and to identify, evaluate, and prioritize alternative desired states (gap analysis). Specific templates facilitate these tasks. The second phase is concerned with competency analysis, defining the new desired behavior, and identifying who will be affected by a change. Plans for motivation, competence, communication, and change are then developed in the third phase, followed by the actual implementation phase where follow-up and learning from the implementation are essential. The model emphasizes the key role of the top manager as a change leader to achieve sustainable organizational and individual behavioral change. It describes factors influencing staff’s willingness to participate in a change process: understanding why the change is beneficial, having influence over the change, knowing how to perform new tasks, and feeling a sense of belonging. These factors are in line with Self-determination theory, which explains how intrinsic motivation to participate in a change is enhanced by focusing on the three psychological needs of mastery/competence, autonomy, and relatedness [21]. In addition, structures and tools helped carry through the change.

The multifaceted implementation strategy relied on target group involvement, dialogues, quick reference guides, informational and educational activities, networking, and audit and feedback [16, 22, 23]. We used the results of the Act in Time pre-implementation studies [14, 15] to refine the strategies. Moreover, we regularly updated managers at all levels on the status of the project.

A main strategy was the use of both IFs and external facilitators (EFs) [24, 25]. The joint role of the facilitators was to guide and support the PHCCs in adopting and sustaining a health-promotion practice. The four EFs had experience in quality improvement work, and three of them had over 25 years of clinical experience and were experts in health promotion. The EFs were trained by the project leader (Y.N.) in change management for this specific project. During the training, all steps of the Astrakan model [20] were carried out along with in-depth discussions of the steps in relation to the project. A step-by-step work plan was developed to execute the different steps of the model. Regular meetings to support the EFs were held with two of the researchers (Y.N. and E.N.S.) during the implementation phase, focusing on sharing experiences and solving potential difficulties.

The EFs worked in pairs and provided structure and support for change to the managers and the IFs. After initial discussions with the respective manager and the IFs, the project was presented at a staff meeting. The EFs held regular meetings, initially weekly, with the IFs and with the managers at each PHCC over at least a 12-month phase. The EFs provided support in conducting a gap analysis, formulating a vision and a goal for the specific PHCC, getting the staff on board, strengthening internal motivation, working with systematic improvement, setting specific, measurable, achievable, relevant, and time-bound goals, and formulating an action plan.

Data on the number of registered health-promotion activities were reported by the controller at the HCA and fed back monthly to the PHCCs by the EFs. Practical support was provided to help staff produce quick reference guides and visualize the clinical intervention process and outcomes; other support was also provided upon request. Networks for IFs and managers were held separately, and the activities in the action plan were followed up regularly. The managers encouraged the staff to participate in digital lifestyle education and included the vision, goal, and outcomes on the agenda for staff meetings. Before withdrawing support, the EFs provided the managers with a sustainability plan. This plan, which was based on published literature, focused on communicating the vision, aim, and goal, organizing the work by using coordinators for each lifestyle habit, and providing routines, tools, educational efforts to maintain competence, and regular follow-up [26–28].

Two to three IFs with knowledge of health promotion, an interest in leading change, and the personal traits and interpersonal skills that would benefit the mission were appointed by each PHCC manager. These IFs worked closely with their manager to achieve the desired state of the PHCC. Based on their knowledge of the PHCC context and their considerations of compatibility with the current way of working, they decided which target groups (diagnoses, professions) to approach, in which order, and in which way, and formulated an action plan. They then carried out the activities with support from the EFs.

### Outcomes

The primary outcome was staff-registered medical record data on the administrative record of simple advice, consultative conversation or qualified consultative conversation (see description in Additional file 1 (A1)., and physical activity prescriptions. The registrations were made by the healthcare professionals. A controller at the HCA provided aggregated de-identified data from the central medical record system from 6 months before the start, during the implementation intervention phase, and 6 months after the end of implementation support.

The secondary outcome was medical record data on standardized screening forms targeting physical activity, diet, tobacco use, and alcohol use. Data on staff turnover for 2022–2023 were requested from the PHCC managers. Potentially influential events during the study period were recorded by two of the researchers (Y.N, E.N.S).

### Sample size

To estimate staff adoption of the new practice, we expected a minimum change from baseline to follow-up of 10%, assuming a base rate (monthly health-promotion activities in controls) of 100 and an estimate of the proportion of variability (seasonal variability etc.) of 20%. For a one-tailed test, 9 PHCCs were required to achieve 80% power (alpha 0.05). We included 10 in total: 5 intervention centers and 5 matched control centers.

### Statistical analysis

Uptake was modeled using generalized linear mixed models, assuming a negative binomial error distribution with a logarithmic link function. Estimation of parameters was performed using maximum likelihood. The response variable was uptake (number of health-promotion activities) in each month. We included the logarithm of the number of visits in each month as an offset term to model rate of uptake.

As the duration of the active implementation phase differed between sites, we defined two time variables: time since start of intervention (time v1), and time since start of post phase (time v2). Both variables were modeled using restricted cubic splines, with three knots placed at the lower, middle, and upper quartiles, and negative values were set at zero for modeling purposes. Accordingly, all baseline months before the start of active implementation were considered time v1 = 0, and time v2 had the effect of modifying the slope of time v1 only during the post phase.

Health-promotion activity (no, yes) was included as a categorical explanatory variable. We modeled calendar month using a restricted cubic spline with three knots placed at the lower, middle, and upper quartiles to allow for seasonal changes. We used a simple linear term for time scaled relative to the earliest month observed in the study to adjust for any long-term trends in uptake rate. Patient’s sex and site pair were categorical terms modeled using dummy variables, and site was included as a random effect.

We pre-specified a series of competing models. These included a full three-way interaction between intervention, sex, and each of time v1 and time v2 (model 1); a reduced form of model 1 with only two-way interactions (model 2); and a further reduced model where intervention was only allowed to interact with each of time v1 and time v2 respectively (model 3). We also assessed whether increasing the number of knots from three to four on the time v1 and time v2 spline terms improved goodness of fit for each of the above settings (models 4–6 respectively), yielding six models in total to compare. Based on comparisons of the Akaike information criterion, model 2 was the best fitting model, and so we selected this for inference. Checks of model assumptions and fit (e.g. dispersion, homogeneity of residuals, autocorrelation) from this model were performed on simulated residuals and are presented in Additional file 2 (A2).

From our selected model, we estimated marginal mean uptake rate (per 1000 visits) and 95% confidence intervals for a typical site with baseline, active, and post phase lengths of 6, 12, and 6 months respectively. These estimates can be thought of as being from a typical site, randomly selected from the population. The estimates were averaged over the levels of pair and sex, with calendar month and time trend held constant at their respective means.

We computed model-based contrasts of the intervention effect with 95% confidence intervals adjusted for simultaneous inference using the multivariate t distribution. The intervention effect (I) on the logarithmic scale is the difference-in-difference estimate:

1 $$log\left(I\right)={\left\{log\left({\mu }_{t}\right)-log\left({\mu }_{t0}\right)\right\}}_{T}-{\left\{log\left({\mu }_{t}\right)-log\left({\mu }_{t0}\right)\right\}}_{C}$$

where $\mu$ is the expected uptake rate, *t* is time since baseline (t0) (i.e. study time v1 = 0), and subscripts T and C denote treatment and control groups respectively. Upon exponentiation to the response scale, this becomes a ratio of rate ratios (RRR):

2 $$I=\frac{{\left({\mu }_{t} / {\mu }_{t0}\right)}_{T}}{{\left({\mu }_{t} / {\mu }_{t0}\right)}_{C}}$$

which we used to present the intervention effect. Due to the longitudinal nature of the study, we present smooth plots of the RRR and simultaneous 95% confidence intervals to visualize temporal changes. Statistical analyses were performed using R, relying heavily on the packages glmmTMB [29], DHARMa [30], emmeans [31], and the tidyverse suite [32].

## Results

All intervention centers completed the active implementation phase, during which 135 002 healthcare visits took place and 8839 health-promotion activities and 2423 lifestyle screening forms were administered. Simple advice, consultative advice and qualified consultative advice were registered for all four lifestyle habits (A1). The corresponding numbers for the control centers were 160 987, 6171, and 282, respectively (Table 1). The mean uptake rate of registered health-promotion activities per 1000 visits during the three study phases was 39.7 (baseline), 65.5 (active), and 136.5 (post) in the intervention centers and 38.6 (baseline), 38.3 (active), and 73.2 (post) in the control centers. Thus, during the active implementation phase, the intervention centers sent out 8.59 times as many screening forms as control centers and demonstrated a crude mean difference in uptake compared to controls that increased from 1.1 per 1000 visits in the baseline phase to 27.2 in the active phase and 63.3 in the post intervention phase. Full data for all matched PHCs per phase and month are presented in Additional file 3 (A3).

**Table 1:** Descriptive statistics for the intervention and control centers in the three study phases

Phase	Healthcare visits		Sent out lifestyle questionnaires		Health-promotion activities		Mean uptake per 1000 visits		Crude mean uptake difference
	Intervention	Control	Intervention	Control	Intervention	Control	Intervention	Control	Per 1000 visits
Baseline	42 436	48 699	32	194	1685	1880	39.7	38.6	1.1
Active	135 002	160 987	2423	282	8839	6171	65.5	38.3	27.2
Post	57 082	67 481	2275	278	7790	4939	136.5	73.2	63.3

Data include all monthly paired primary healthcare center comparisons; these comprised 21 comparisons from baseline, 64 from the active phase, and 39 from the post-intervention phase (see Supplementary Information 2). The summer months (June, July, and August) were excluded from presentation

**Fig. 1:**
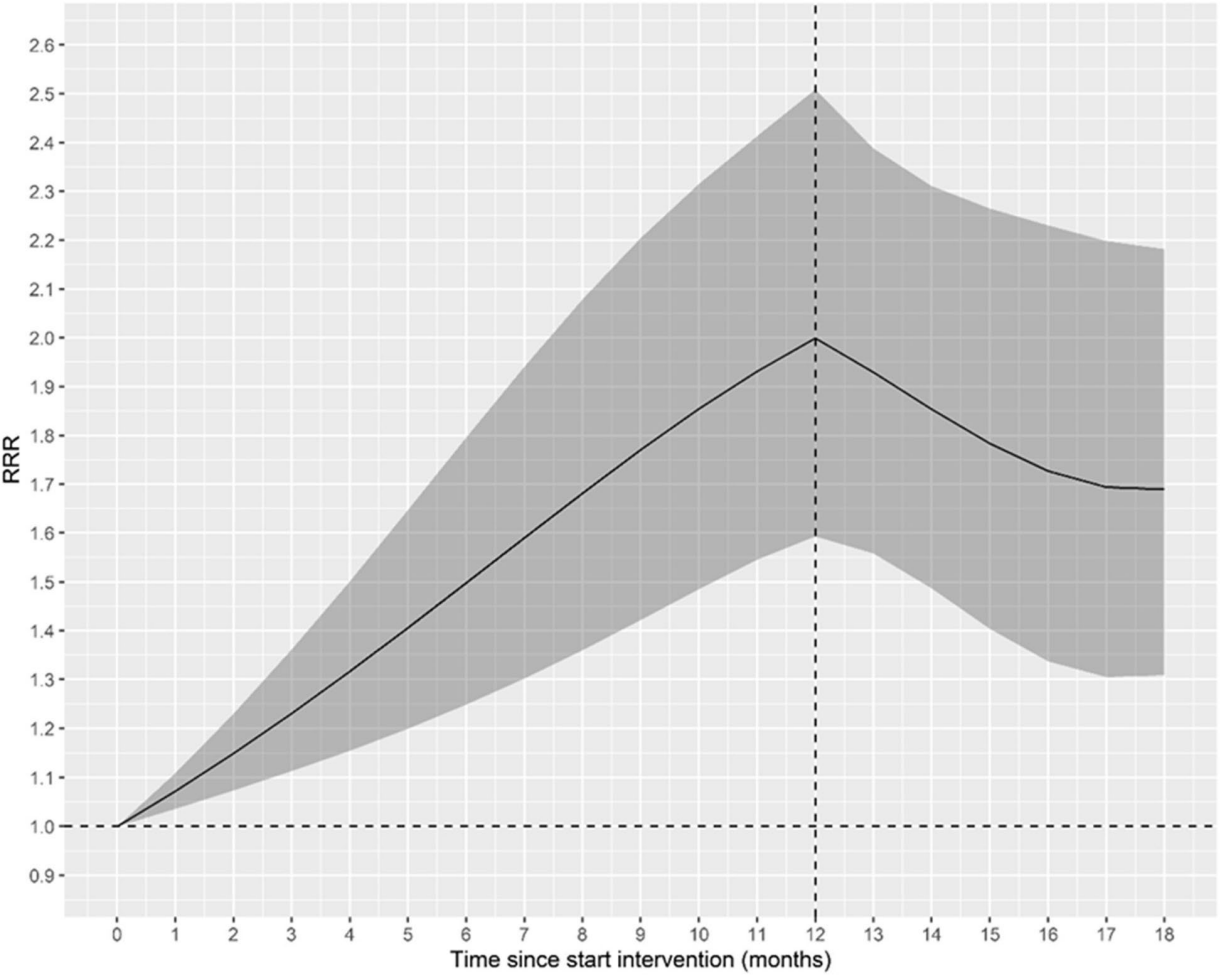
Relative intervention effect over the 12-month active implementation phase and the 6-month post-intervention phase. Solid line: rate of rate ratios; shaded area: 95% confidence intervals

To estimate the effect of the clinical intervention for inference, uptake was modeled using generalized linear mixed models. Figure 1 shows the relative intervention effect during the 12-month active phase and the 6-month post-intervention phase. The RRR increased approximately linearly during the active phase so that, on average, the rate of uptake of health-promotion activities at the intervention sites was 1.07 and 2.0 times the rate at the control sites at 1 and 12 months, respectively and are provided in Additional file 4 (A4). The intervention effect was also apparent in the post phase, although the magnitude was slightly reduced; at 6 months into the post-active phase, the intervention centers had approximately 1.69 times the uptake rate of the control centers.

**Fig. 2:**
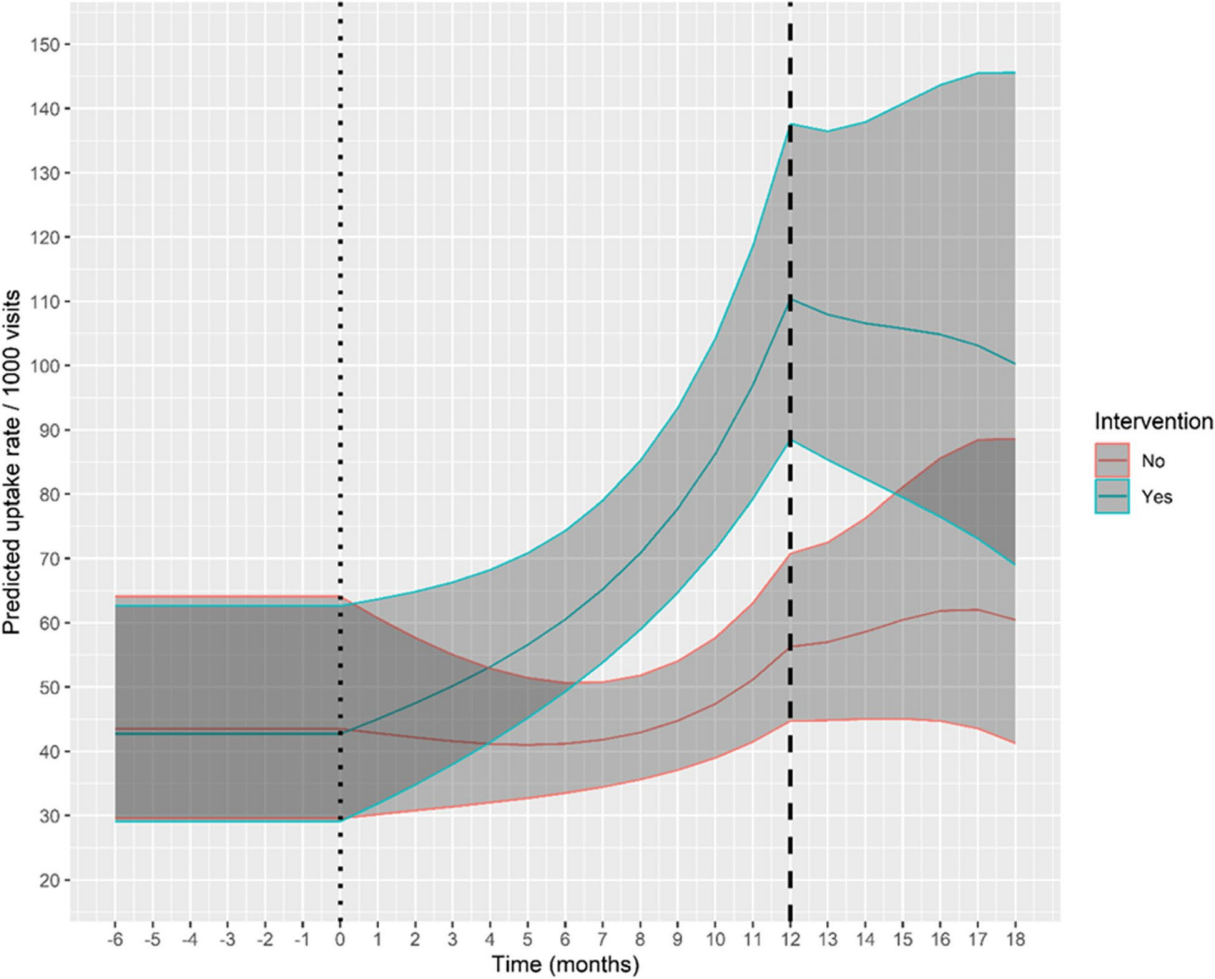
Model-based predicted mean uptake rate for health-promotion activities for the intervention and control sites with baseline, active, and post-phase lengths of 6, 12, and 6 months, respectively

Figure 2 shows the model-based predicted mean uptake rate of health-promotion activities for the intervention and control sites with baseline, active, and post-phase lengths of 6, 12, and 6 months, respectively. In the intervention group, the uptake increased exponentially during the active phase. Seven months into this phase, an absolute effect of a 23.41 higher uptake rate of health-promotion activities per 1000 visits was predicted compared with controls, with confidence intervals excluding unity in months 7–14. The confidence intervals are shown in the Additional file 5 (A5). By the end of the 12 months of implementation support, the difference in uptake rate had increased to 54.09 per 1000 visits. The effect then leveled off in the post-implementation phase, although the confidence intervals do not rule out a plateau effect in this phase.

The effect of the implementation strategy on uptake rate over time did not vary by patient sex for either the active phase (χ^2^ = 2.5056, *p* = 0.2857) or the post phase (χ^2^ = 1.9971, *p* = 0.3684).

### Influencing events

Control centers had a similar number of staff (*n* = 277) as the intervention centers (*n* = 280) in 2022 and 2023, but the intervention centers had lower staff turnover (15%) than the control centers (26%). The COVID-19 pandemic, which reached Sweden in 2020, had a major impact on healthcare services. Most of the restrictions and general advice directed to the population due to the pandemic were lifted in February 2022. The present study started when restrictions were still in place, but the peak of infected people needing healthcare had passed. For most of the implementation phase, meetings were held digitally. A reorganization was announced in January 2021, which created a dilemma in informing managers, as all managers were dismissed and encouraged to apply for the new, smaller set of managerial positions. Some IFs were replaced during the study phase.

## Discussion

The implementation strategy in the present study was successful in achieving a change towards a more health-promoting practice. This was demonstrated by intervention sites administering 8.59 times as many lifestyle screening forms as control sites, having a higher relative uptake that was sustained through the 6-month post-intervention phase, and having an absolute effect (i.e., the group difference in the predicted uptake rate per 1000 visits) that was evident 7 months into the active phase. Improved health promotion in PHC means that more people can reflect on their health behavior, gain knowledge of why and how to address unhealthy lifestyle habits, and in the extension, likely gain more health. Crude data are presented in addition to relative and absolute effects but should be interpreted cautiously since there were significant differences in this uptake between the matched pairs as shown in A3.

The A3 shows that in some cases, control centers had more health-promotion activities including lifestyle questionnaires at baseline. Despite this, the implementation strategy resulted in a higher uptake in the intervention centers during the study period. Sending out questionnaires prior to visits provides an opportunity to triage (i.e. to assess and prioritize to whom advice or counseling should be addressed) and thereby work more efficiently. While observing a patient in real life can provide some information on unhealthy lifestyle habits, not all such habits are visible. It is possible that the staff had more health-promotion discussions with patients than were registered in the medical records. However, this possibility is the same for both intervention and control centers.

We aimed for a bottom-up implementation approach, to build a sense of ownership of the process, with strong leadership support and consideration of inner contextual factors. The implementation strategy was therefore tailored by information collected in the pre-implementation phase, which assessed barriers and facilitating factors [14] and staff’s expectations of and readiness for change [15] using multi-professional focus group discussions and individual interviews with managers and IFs. Our impression is that these initial discussions and interviews initiated the uptake process. The manager and the IFs formed an in-house team that enabled leading change from within using a bottom-up perspective. Combining this bottom-up approach with a top-down perspective through anchoring the project with the CEOs showed that health promotion was prioritized. Top managers are important in allocating the resources needed for an implementation process [33]. In the present study, the manager of the Unit of Development allocated EFs to support the implementation.

The PHCC managers were ultimately responsible for the change process and communication about the change [20], while the IFs were merely responsible for supporting the change. This interdependent relationship between managers and IFs may have helped or hindered the implementation, and we will explore this further in individual and group interviews. It has previously been suggested that interactions between managerial leaders and facilitators are characterized by realizing commitment, negotiating conditions, and encouragement to maintain momentum [34]. There is a fine line between delegating and relinquishing responsibility and authority. The managers remain responsible for the change and for communicating the change.

Knowledge of health-promotion practice and the local organization was an advantage gained through appointing the IFs. These IFs were not required to have change management experience when appointed but were trained in change management techniques. The managers selected their IFs and were therefore able to choose individuals in whom they had confidence and who had the desired interpersonal skills. By appointing them, they gave the IFs credibility in their role and as individuals, which presumably led to respect from staff. Vulnerability was reduced by appointing at least two IFs per center. The study information clarified that the facilitators should be given dedicated time for their work.

The role of EFs in helping others to change their clinical practice [35] appears to be of great importance in maintaining a focus on health-promotion practice in an interchangeable world, and has been found to be associated with successful implementation [36]. There is no consensus on how IF training and support should be provided. In the current study, EF support and IF training were rigorous throughout the study. Sharing of experiences has been suggested to be important [24], and in the present project was achieved through networking led by the EFs. As the PHCCs started the implementation at different times, those that started later were able to benefit from the experiences of their counterparts that started earlier. The EFs facilitated meetings and networking, inspired and encouraged the IFs, gave examples of how to use storytelling in communication, and provided practical support when producing quick reference guides and making slide presentations.

The presence of comparison centers was crucial because the overarching operational plan for the healthcare administration in the Region Örebro clearly states that all PHCCs should work with health promotion. This implies an expectation that all PHCCs in the region should gradually improve in this regard. Our findings illustrate that even if goal setting in operational plans is important in providing direction, these goals should preferably be accompanied by practical support to achieve them. The current implementation strategy was based on a shared belief in the importance of health-promoting practices in PHC and a willingness to allocate resources, including staff and time, to work towards this goal. The intervention and control centers had a similar number of staff, but the control centers had higher staff turnover. This is often a source of stress for an organization and might have been a reason for these centers’ lack of interest in becoming intervention centers.

The project drew attention to health-promotion practice during the study period, and information about the project was reported on several occasions at meetings attended by all PHCC managers. This probably resulted in some dissemination of the implementation strategy to the control centers, introducing contamination of the results. Unpredictable external and internal events inevitably affect clinical research projects. The reporting of influencing events puts the implementation efforts in a real-world context and facilitates the understanding of the conditions.

### Limitations and strengths

The criterion that only PHCCs willing to receive structured support from the project to achieve change were included as implementation centers may have introduced selection bias. However, this inclusion criterion relies on leading change theories emphasizing that change starts with a sense of urgency and a cognitive dissonance evolved by realizing the gap between the current and the desired state [20, 37]. The energy and power to change are fueled by a so-called threat image and a vision. It would not be possible, nor beneficial, to force structured support on PHCCs that wanted to continue on their own to achieve a more health-promoting practice. Moreover, we have no data on the quality of the health-promotion activities provided by the centers; that is, on fidelity to the recommendations. Nevertheless, the context-tailored support enhanced the ability to respond and adjust the support to PHCC-specific conditions. A strength of the study was the support for the implementation of this project given by the higher levels of management.

To our knowledge, implementation studies on health promotion are seldom quantitative, and rarely use implementation strategies directed to all staff members. Instead, district nurses are often the informants in qualitative studies. Involving all staff strengthens the relatedness and is likely to be important in changing clinical practice. Randomized controlled trials (RCTs) are the gold standard for evaluating intervention effectiveness and establishing causality, prioritizing internal over external validity. However, non-RCTs can play an important role in real-world settings, especially when implementing evidence-based interventions. The present study design was not only longitudinal, but also included parallel comparisons, using matched controls, over a total of 24 months of complete data. We also incorporated covariate adjustment as well as advanced modeling techniques such as regression splines for capturing non-linear associations. We included testing of model assumptions and fit as illustrated in the A2. However, in the absence of randomization, we are unable to rule out selection bias, and therefore some caution is warranted. Nevertheless, we anticipate that our study will be the catalyst for future enquiry, particularly randomized studies, to rigorously assess causal associations and generalizability. We therefore consider the design to be relevant. We used the Standards for Reporting Implementation Studies (StaRI) [38].

Healthcare is a complex organization that includes many professional roles, and implementation projects may introduce additional roles, such as facilitators. Further research on how staff in different professional roles experience the implementation of a complex intervention may provide information that can be used for knowledge translation. Sustainability over a longer period will be assessed quantitatively in the current project, supplemented by individual interviews with managers at different levels to gain an understanding of what influenced sustainability from their point of view.

## Supplementary Information

Additional file 1: Description of advice levels (A1)

Additional file 2: Checks of model assumptions and fit (A2)

Additional file 3: Descriptive statistics by matched Primary Healthcare Centers (A3)

Additional file 4: Rate of rate ratios (RRR) and lower and upper 95% confidence limits per study month (A4)

Additional file 5: Predicted monthly uptake rate per 1000 visits by study group and lower and upper 95% confidence limits (A5)

Additional file 6: StaRI checklist

## Data Availability

The datasets supporting the conclusions of this article are included within the article and its additional files.

## Abbreviations

CEO: Chief executive officer
EF: External facilitator
HCA: Healthcare Choice Administration
IF: Internal facilitator
IHA: Integrated Healthcare Administration
PHC: Primary healthcare
PHCC: Primary healthcare center
RCT: Randomized controlled trial
RRR: Rate of rate ratios
