# Supplementary material for: Achieving health-promotion practice in primary care using a multifaceted implementation strategy: a non-randomized parallel group study
Source: Implement Sci Commun. 2025 Apr 7;6:36. doi: 10.1186/s43058-025-00723-y (PMC11977894; doi:10.1186/s43058-025-00723-y)
Supplement: Supplementary file 1 — Additional file 1: Description of advice levels (A1) [file 43058_2025_723_MOESM1_ESM.docx]

**Description of level of advice for health-promoting activities**

| **Level of advice** | **Description** |
| --- | --- |
| **Simple advice** | Constitutes a self-evident part of an assessment interview at all levels of healthcare. Verbal information with short, standardized advice and recommendations, possibly completed with written information. Potential referral to another instance. |
| **Consulting conversation** | A dialogue with the patient, adjusted to the patient’s prerequisites (for example age, health and risk factors).  Includes motivational strategies when needed and  different tools and aids may be added. Follow-ups on one or more occasions are often offered. |
| **Qualified consulting conversation** | Besides what is mentioned for consulting conversations – the dialogue should be held in a structured manner, based on behavior change theory. The professionals should be deeply knowledgeable about the current lifestyle habit. |
