# Supplementary figures and images for: Achieving health-promotion practice in primary care using a multifaceted implementation strategy: a non-randomized parallel group study
Source: Implement Sci Commun. 2025 Apr 7;6:36. doi: 10.1186/s43058-025-00723-y (PMC11977894; doi:10.1186/s43058-025-00723-y)

**Additional file 2 (A2).**

Checks of model assumptions and fit.


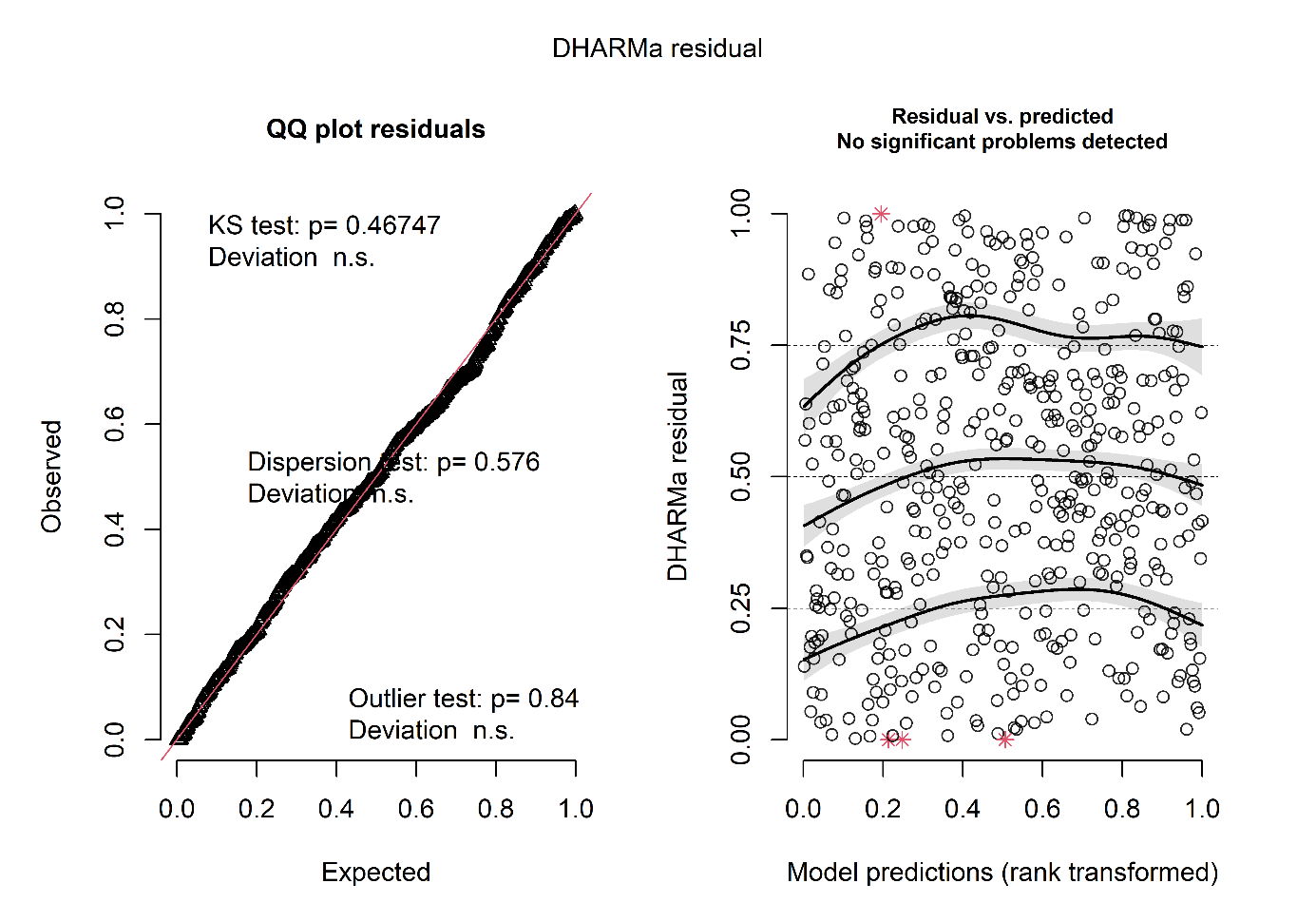

Supplement: Supplementary file 2 — Additional file 2: Checks of model assumptions and fit (A2) [file 43058_2025_723_MOESM2_ESM.docx]
